# Analysing Institutions Interdisciplinarity by Extensive Use of Rao-Stirling Diversity Index

**DOI:** 10.1371/journal.pone.0170296

**Published:** 2017-01-23

**Authors:** Lorenzo Cassi, River Champeimont, Wilfriedo Mescheba, Élisabeth de Turckheim

**Affiliations:** 1 Observatoire des Sciences et Techniques, HCERES, Paris, France; 2 Paris School of Economics, Université Paris 1, Paris, France; 3 INRA, Délégation à l’Évaluation, Paris, France; Universidad de las Palmas de Gran Canaria, SPAIN

## Abstract

This paper shows how the Rao-Stirling diversity index may be extensively used for positioning and comparing institutions interdisciplinary practices. Two decompositions of this index make it possible to explore different components of the diversity of the cited references in a corpus of publications. The paper aims at demonstrating how these bibliometric tools can be used for comparing institutions in a research field by highlighting collaboration orientations and institutions strategies. To make the method available and easy to use for indicator users, this paper first recalls a previous result on the decomposition of the Rao-Stirling index into multidisciplinarity and interdisciplinarity components, then proposes a new decomposition to further explore the profile of research collaborations and finally presents an application to Neuroscience research in French universities.

## Introduction

Interdisciplinarity refers to complex processes of knowledge production that involves cognitive dynamics as well as a social construction [1–3]. It also covers a variety of ways to confront and bridge disciplinary approaches. Among the various definitions and terms that have been proposed in a now long standing debate, the distinction between multidisciplinarity, as a juxtaposition of disciplinary components, and interdisciplinarity which integrates knowledge, theories or methods from different disciplines is widely shared [4]. However, there is not a single way to characterize interdisciplinarity as one could consider the degree of disciplinary integration, the interdisciplinary practices or the rationales of interdisciplinarity [5, 6]. Understanding interdisciplinarity therefore deserves combined methods involving an analysis of the integration process as well as quantitative measurements based on outcomes [1, 3]. Among quantitative methods, the bibliometric literature proposes different indicators, based on different data, different levels of aggregation and different visualisations adapted to different needs of indicators users.

As interdisciplinary research is expected to be a powerful factor in the renewal of science and of its societal effectiveness, many research institutions are now promoting it. It is the case in France where a process of consolidating and merging universities has been under way for ten years, strongly encouraged by the government. This process aims to improve the international visibility of universities, to strengthen synergies between local institutions and, more generally, to improve the effectiveness of the research system. A new institutional proximity between research teams is often seen as an opportunity to develop new interdisciplinary research programmes. Universities management teams are therefore looking for new indicators to identify the potential of interdisciplinarity and to follow-up their strategies and the impact of incentive measures they have implemented.

At the Observatoire des Sciences et Techniques of the French High Council for Evaluation of Research and Higher Education (HCERES-OST), effort is being put in to provide indicators that are appropriated by end users and to deliver new indicators for research institutions that are often supported by training sessions. To introduce information about interdisciplinarity in the universities set of monitoring indicators, OST selected the Rao-Stirling diversity index calculated on the distribution of WoS categories of references in publications. This choice was made because of the integrative property of this index [7] and because it is based on the same data as the usual set of indicators provided by OST (the OST enriched version of the Thomson Reuters databases). Moreover, the ability of the Rao-Stirling index to cope simultaneously with two levels of interdisciplinarity—publication level and department level— [8] is adapted in a context where universities are in an exploratory phase rather than considering precise hypotheses about interdisciplinary dynamics.

In order to test the understanding and to improve the ergonomics of this indicator, a group with members of management teams of French universities has been set up. This interaction with OST resulted in a better description of what the indicators measure, as well as new graphics to position universities interdisciplinary orientations in the landscape of national or international research in a specific scientific field. Consequently a specific field had to be chosen and Neuroscience emerged as a good candidate given its multidisciplinary nature and the scientific profiles of the universities involved in the working group. Finally, computer programs were consolidated and made freely available for users [9].

This paper presents the whole method, that is including a review of the properties of the Rao-Stirling index and of its decomposition. It therefore partly overlaps the previous paper [8] but this aims at facilitating the usage of this bibliometric approach of interdisciplinarity. The application to the measure of interdisciplinarity of eight French universities in Neuroscience illustrates the possible use of this bibliometric approach to provide a comparative analysis of the cited disciplines by the institution publications and to raise issues about institutions specific scientific strategies.

## The Rao-Stirling index and its decompositions

### Defining the index, choosing the classification and the distance

If interdisciplinarity is considered as the integration of empirical material, methods or theories from different disciplines, a simple way to capture a part of this integration is to track the diversity of influences on a particular scientific work as the disciplinary diversity of the documents cited in publications. Diversity indexes are based on a classification and usually accounts for the number of classes in which items are present—the variety—and for the balance of the distribution of items into classes. When measuring interdisciplinarity, the choice of a classification of publications into discipline categories is related to the type of interdisciplinarity under study, and whether close interdisciplinarity should be accounted for as well as remote interdisciplinarity. Some authors [10–12] calculate indicators and analyse interdisciplinarity for two levels of a hierarchical classification of science. Another way to cope with this issue of the disparity of disciplines is to integrate a distance in the definition of the diversity index. The Rao-Stirling index, which is now widely used in interdisciplinarity studies [13–16], takes into account variety and balance but also a distance between categories in such a way as to give a greater weight to pairs of references in more distant categories [7]. This index is defined as follows


$$\begin{array}{c} ST=\sum_{i,j}q_{i}q_{j}d_{ij} \end{array}$$

where *q*_*i*_ and *q*_*j*_ are the proportions of references in categories *i* and *j* and *d*_*ij*_ is the distance between the two categories. This index integrates close and remote interdisciplinarity by weighting them in a single formula. It is interesting to notice that this index is robust to an over specification of categories so that the choice of the classification less problematic. This is because the overall index does not change much when two categories with a small distance are merged into a single one. More precisely, the variation *D*(*ST*) of the index *ST* when category 1 and 2 are merged is such that


$$D\left(ST\right)<d_{12}\left(q_{1}q_{2}+\max\left(q_{1},q_{2}\right)\right).$$

In a way, this partly defers the issue of carefully choosing the classification to that of choosing the distance. Different distances designed to achieve graphical maps of science may be used in a diversity index. We use the distance which is derived from the cosine similarity between the profiles of references of the two categories in the whole database (i.e. the inter-category aggregated citing dimension) [17, 18].

### Normalizing the indicators

This basic index is used to design indicators for different analyses. As the citation practices of scientific communities are different and as the databases do not cover the various scientific fields—or disciplines or subject categories—homogeneously, the index has different ranges for different fields. This introduces uncertainty into the interpretation of the comparison between institutions. An institution could have a high indicator value not truly reflecting a higher propensity towards interdisciplinary research but only because it is specialised in scientific fields in which the cited references of papers are covered more exhaustively as source items in bibliographic databases, or in fields in which researchers can include, on average, more references per papers due to editorial rules or to subfield-specific citation practices. Campbell and co-authors [15] tested various correction methods to account for different citation practices as well as coverage biases at the sub-field level for applications where it might be necessary to perform aggregated comparisons (all fields combined) at the level of institutions, regions (states/provinces) or even countries. However, these corrections all come with some limitations, the main one being a severe reduction in the number of publications which are retained in the analysis (for a deeper discussion of their approach, see [15]). Where possible given the goal of the study, another and more pragmatic way to cope with this issue is to perform separate analyses for the different research fields in which an institution is active. This is the approach we recommend and apply here. This choice raises the issue of a research field delineation. In order to make comparisons possible, equivalent corpora should be selected for the various institutions. The easiest way is to select a set of journals and consider all publications in these journals in the chosen period of time. This also allows to use centred indicators—i.e. difference between the institution indicators and the world indicators, which range between 1 and -1 with a symmetrical scale on both sides of 0.

### Breaking down the indicator into interdisciplinarity and multidisciplinarity components

Many authors calculate the interdisciplinarity index of an institution as the average of the interdisciplinarity indexes *ST*_*a*_ of publications [13] [15]. The Rao-Stirling index of a paper is a proxy for the integration of disciplinary sources of the work reported in a paper and their average over the publications of an institution or of a department is a proxy for the degree of interdisciplinarity of the department. We denote it *ST*_*W*_ and refer to it as the *within* index [8]. However, interdisciplinarity is a social process and the cultural and management environment in a department plays a role in the effective or potential interdisciplinary behaviour of the researchers [2]. An indication of an environment that is favourable for interdisciplinarity is partially captured by the diversity of the whole set of references in the department publications regardless of the citing paper. As Garner and co-authors [19], we think that the “integration score for a set of papers (e.g. by ‘project’) provides an additional perspective” (p 137). It is also the choice that Rafols and co-authors [20] consider relevant for comparing different departments in Business and Management schools and Innovation studies units in the UK. This approach leads to calculate an overall diversity index denoted *ST* based on the whole list of references of a department publications which value is higher than the *within* index *ST*_*W*_.

An interesting property of the Rao-Stirling index is its similarity with the inertia of a set of weighted points. If a point is associated with each pair (*a*, *i*) where *a* is an article and *i* is the category of one of its references and if the distance between two pairs (*a*, *i*) and (*b*, *j*) is *d*_*ij*_ and if at last *d*_*ij*_ is the square of an Euclidean distance, then the overall index is exactly the inertia of the set of points (*a*, *i*) [8]. We choose a weight for each pair (article, category) which is the proportion of references in the category for the given article and, in the formula for the overall index, *q*_*i*_ is then the average of the proportions of category *i* in the articles. This choice gives the same weight to each article and ensures that articles with a very large number of references as review papers will not impact too much the overall index. This is not be true if the same weight is given to any reference whichever the number of references in the citing paper (see [8] for this other option).

Considering the references of each individual publication as subsets of the whole set of references and applying the usual decomposition of an inertia into its within group and between group components—an application of Pythagoras theorem—allows to decompose the overall index into two components, the first being the average of the Rao-Stirling indexes of articles


$$ST_{W}=\frac{1}{n}\sum_{a=1}^{n}ST_{a}\;.$$

The difference *ST*_*B*_ = *ST* − *ST*_*W*_ captures the variation of the sources between publications and this second index can be written as the inertia of the set of *n* points, each point representing the centre of gravity of the references of an article. As we interpreted the *within* index *ST*_*W*_ as the effective interdisciplinarity, the other component would be a non-integrated component of the overall diversity or a residual diversity which is a proxy for the thematic diversity of the publications of the department, corresponding to juxtaposed disciplinary perspectives in the institution but without integration in published work. We therefore suggest to use the terms interdisciplinarity and multidisciplinarity for the two components of the overall index. However, this use of the two terms is not consistent with the usual definition where multidisciplinarity and interdisciplinarity refer to juxtaposition *versus* integration of disciplinary contributions in a selected piece of research as a single publication or a set of publications on a given topic. [3].

This decomposition of the overall diversity index generates four patterns summarised in Table 1.

**Table 1:** Four types of disciplinary diversity (adapted from [8]).

	**Interdisciplinarity** index	
	*lower than* *world benchmark*	*higher than* *world benchmark*
**Multidisciplinarity** index		
*higher than* *world benchmark*	4. Thematic diversity with specialised articles	1. Thematic diversity with integrative articles
*lower than* *world benchmark*	3. Niche research with specialised articles	2. Niche research with integrative articles

A two dimensional graphical representation of institutions along these two components of interdisciplinarity provides a more quantitative information. The four quadrants in such a representation correspond to four types of disciplinary diversity, combining specialisation *versus* integration at the publication level (on the *ST*_*W*_ axis) with thematic diversification *versus* thematic concentration at the institution level (on the *ST*_*B*_ axis). This decomposition of the overall index thus provides some differentiation between institutions with similar overall indexes.

### Breaking down the indicator into specific profiles

When the overall index of a corpus is higher than the world index, a natural question, specifically asked by the user group of universities, is to know which categories are cited more by the institution than the standard world behaviour, and which are cited less. To answer this question, it is possible to directly compare the proportions of references between universities. Unfortunately these different of proportions will not be related with the overall index because they do not account for disparity between the cited references. A discipline more cited by a university should be weighted more if the discipline is distant from the set of other references. A consistent way to weigh disciplines is to break down the overall indicator as a sum of category contributions. The contribution *C*_*i*_ of category *i* is therefore defined as the product of the average proportion *q*_*i*_ of references in the category and the average distance of this category to the other categories of references ∑_*j*_ *q*_*j*_*d*_*ij*_ so that


$$C_{i}=q_{i}\sum_{j}q_{j}d_{ij},$$

and the overall index is just the sum of the category contributions


$$ST=\sum_{i}C_{i}.$$

Comparing the category contributions for institutions provides information on the specificity of the institution references profile and insight on causes of a high or small value of the overall indicator.

### Ancillary statistics

In order to derive relevant results from the comparison of indicator values and prevent abusive interpretation when these values could be overly affected by errors or exceptional data, some statistical properties of the indicators are helpful. Considering a multinomial probabilistic model for the reference counts in categories and using the delta-method (a first order approximation of the indicator considered as a function of averages of independent, identically distributed variables) allows to calculate the variance of the indicators [8]. As a central limit theorem applies for the random variables associated to the indicators, it is possible to calculate confidence intervals for each index—overall, *within* and *between*—as well as for the contribution of each category. This normal approximation of the indicators makes it straightforward to know, for any chosen threshold of error—or significance level—if an observed difference between two universities or between a university and a benchmark value is significant or not. This provides a statistical criterion useful to avoid interpreting indicators calculated on samples that are too small or with too much variability. For more information on the calculation of statistical indicators, see S1 Appendix.

## An application to a research field: Neuroscience in French Universities

Neuroscience—also referred to as neurosciences—is the study of the structure, functions, development, abnormalities of the nervous system and its impact on behaviour and cognitive functions both in the normal functioning and in the case of neurological, psychiatric and neurodevelopmental disorders. Neuroscience is currently a multidisciplinary field involving biomedical sciences—such as clinical neurology, psychiatry, cognitive and behavioural science -, fundamental biology—such as genetics and molecular biology -, as well as other disciplines as psychology, linguistics and philosophy, together with engineering, chemistry, physics, mathematics and computer science. Due to the large range of disciplines in the field, it is interesting to measure the diversity of an institution research projects in neuroscience and the level of integration by neuroscientists of theories or methods from different disciplines.

For the bibliometric approach, a corpus representing the domain has to be defined. WoS has a category and Scimago has a subject area with the title Neurosciences whereas the Neurology & Neurosurgery sub-field in Science-Metrix classification does not encompass all relevant literature as defined above (for example some neuroscience thematics are covered in other subfields such as Psychiatry and Clinical Psychology). The WoS Neurosciences category is defined by a set of 255 journals of which 25% of the articles are in journals assigned only to this category, and 27% are simultaneously assigned to Neurosciences and to Clinical Neurology (Data for 2012). The Scimago Neurosciences subject area contains 509 journals, distributed in 9 subject categories. The two sets have 206 journals in common. Selecting these 206 journals could be a relevant choice but, for the sake of convenience, we simply selected the publications in the WoS Neurosciences category as the corpus for this study.

### Positioning universities on three interdisciplinarity scales

Twenty seven French universities produced more than 100 WoS indexed publications in the period 2008–2012. Eight universities are included in this study and among them, the three universities with the highest production in Neuroscience. The other universities were chosen because they participated in the working group. The overall, *between* and *within* interdisciplinarity centred indexes and their standard errors are displayed in Table 2. Student’s t-tests for the difference of indicators between two universities and between a university and the whole France, derived from the standard error of each indicator are shown in Table 3.

**Table 2:** Interdisciplinarity centred indicators and their standard errors for 8 French universities, Neuroscience, 2008–2012.

University	nb of papers	ST	Std error	STW	Std error	STB	Std error
PARIS 6	1391	-3.309	0.409	-1.675	0.294	-1.634	0.266
PARIS 5	1030	1.012	0.483	-0.199	0.353	1.211	0.359
AIX-MARSEILLE	993	-5.961	0.542	-3.739	0.368	-2.222	0.340
PARIS 7	526	2.835	0.650	1.495	0.486	1.340	0.512
STRASBOURG	416	-3.752	0.668	-1.720	0.482	-2.032	0.483
GRENOBLE 1	280	-0.588	0.974	0.830	0.676	-1.419	0.632
BOURGOGNE	158	0.539	1.244	0.431	0.914	0.108	0.837
NANTES	134	6.056	1.236	2.829	0.854	3.227	1.090
FRANCE	8446	-1.336	0.182	-1.402	0.124	0.067	0.127

Indexes multiplied by 100

**Table 3:** Statistics for the comparison of universities interdisciplinarity indicators.

Pair comparison	Diff ST	Z-score	Diff STW	Z-score	Diff STB	Z-score
PARIS 7-STRASBOURG	6.587	8.137	3.215	4.695	3.372	4.787
PARIS 7-PARIS 5	1.823	1.544	1.694	2.820	0.129	0.215
PARIS 5-STRASBOURG	4.764	5.782	1.521	2.507	3.243	5.488
STRASBOURG-AIX M	2.209	2.568	2.020	3.330	0.190	0.321
PARIS 6-FRANCE	-1.973	-4.405	-0.272	-0.853	-1.701	-5.772
PARIS 5-FRANCE	2.348	4.551	1.203	3.214	1.144	3.008
AIX M-FRANCE	-4.625	-8.084	-2.337	-6.020	-2.288	-6.310
PARIS 7-FRANCE	4.171	6.180	2.897	5.776	1.273	2.412
STRASBOURG-FRANCE	-2.416	-3.491	-0.317	-0.637	-2.099	-4.199
GRENOBLE 1-FRANCE	0.747	0.754	2.233	3.248	-1.486	-2.305
BOURGOGNE-FRANCE	1.875	1.492	1.833	1.987	0.042	0.049
NANTES-FRANCE	7.392	5.918	4.232	4.903	3.160	2.879

A z-score is distributed as a normal variable so that |*z* − *score*| > 2.575829 is significant at level 1% (bold in the table)

**Fig 1:**
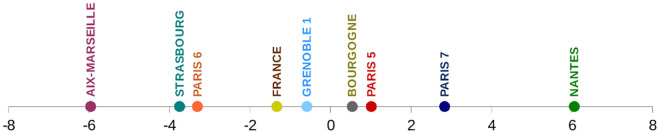
Overall Interdisciplinarity index for 8 French universities. Indexes are centred on the world indexes and multiplied by 100.

The overall index provides a first ranking of the eight universities (Fig 1): the two universities with the largest production, Paris 6 and Aix-Marseille have negative overall index as well as Strasbourg, all three being lower than the national value of the index with statistical error less than 0.1. On the other side, Paris 5 and Paris 7 have a larger index than France. As a small production leads to a large standard error, indexes for Grenoble 1 and Bourgogne are not significantly different from France index, while Nantes index is highly significant indicating a specific position of Neuroscience among the research themes of this university.

**Fig 2:**
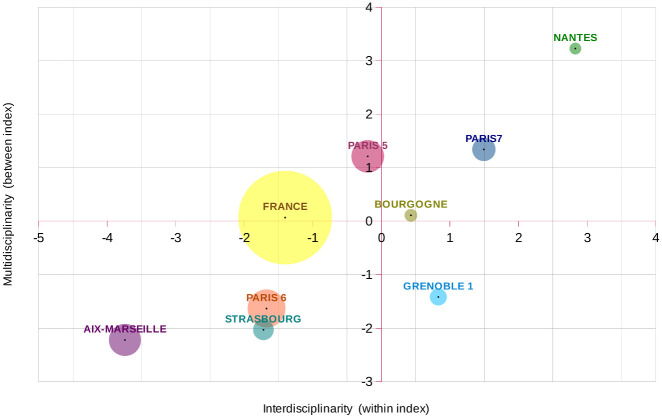
Within and Between Interdisciplinarity indexes for 8 French Universities. Indexes are centred on world values. Bubble sizes are proportional to the number of publications.

Considering the two *within* and *between* components of interdisciplinarity, the ranking of the eight universities is confirmed since the points in the 2D representation are roughly aligned along the bisector (Fig 2). However, this particular configuration does not result from a theoretical positive correlation between the two indexes. For other fields, they are points in other quadrants producing different orders of universities depending on the chosen index (six other examples are shown in S1 Fig). The new information provided by this decomposition is that Paris 7 University high overall index is mainly due to its *within* component, which is larger than France and Paris 5 *within* indexes, indicating an actual discipline integration, while its *between* or multidisciplinary component is more similar to that of Paris 5. The difference between Strasbourg and Aix-Marseille universities is also essentially a difference of the *within* component.

**Fig 3:**
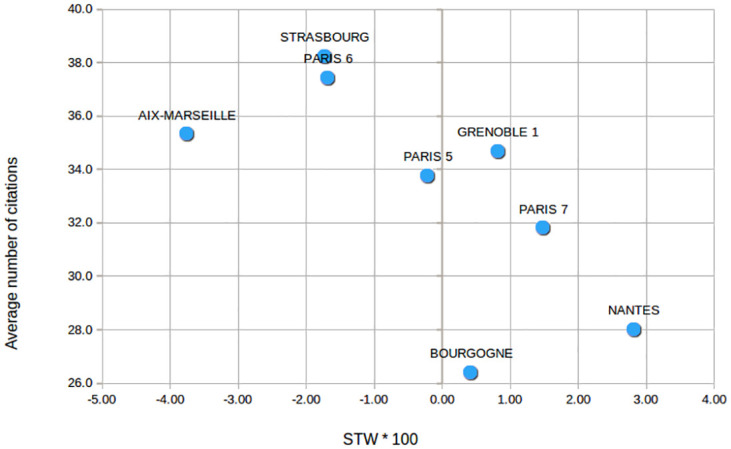
Average number of citations for University publications related with their *within* interdisciplinarity index. A fixed citation window of three year is chosen for citation counts. Citation counts are available in S1 Table.

It is challenging to understand why universities renowned for their Neuroscience research such as Aix-Marseille and Paris 6 have a lower interdisciplinarity index than five of the six other universities. A comparison of this indicator with the average number of citations shows an inverted U-shape functional relationship between the mean number of citations and the *within* index (Fig 3)—with Bourgogne as an outlier point. Calculated on aggregated values and on a small sample of institutions, this observation does not provide any evidence for large scale results, but it is consistent with more general and more careful analyses of the relationship between impact and interdisciplinarity carried out by Larivière and Gingras [10] with another measure of interdisciplinarity—the proportion of citations to disciplines different from the citing article—or by Yegros-Yegros and co-authors [6] with indicators measuring separately the three different dimensions of diversity. However this relationship between citation counts and interdisciplinarity does not explain how interdisciplinarity—as measured by a chosen indicator—occurs, nor is it strictly necessary. To examine more precisely why an index is high or low, it is useful to analyse other information about the publications and about the research organisation in each university.

### Specific university profiles of references

**Fig 4:**
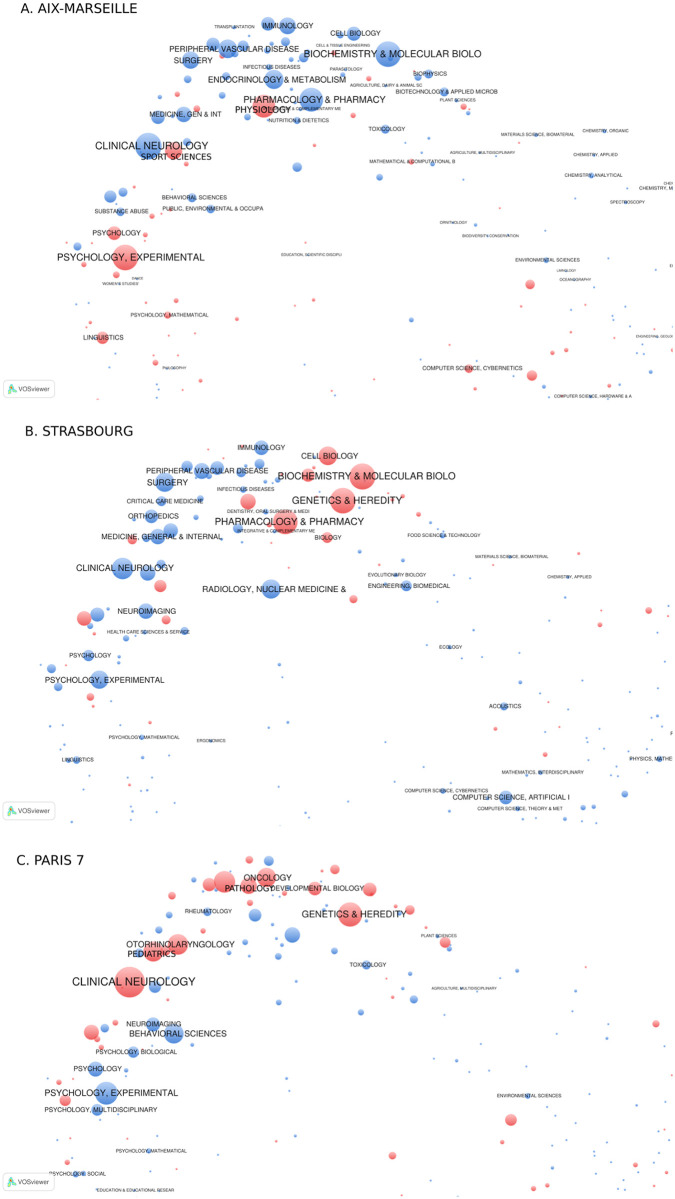
Overlay maps of reference categories in Neuroscience publications A: Aix-Marseille, B: Strasbourg and C: Paris 7. Bubble sizes are proportional to the absolute value of the (world centred) contribution of each category, red when the university contribution is larger than the world, blue if it is smaller. Categories are labelled if their contribution is significantly different from the world (level 1%). Input data for VOSviewer in S2 Table.

To show how to use the information provided by the decomposition of the index into category contributions, we decided to focus on three universities with an important output and showing very different situations: Aix-Marseille, Strasbourg and Paris 7 universities. Results are presented in Table 4. Overlay maps provided by VOSviewer [21] show category weights for each university on a common base map (Fig 4). This base map of categories is obtained with Saltons’ cosine between citing profiles of categories (see S2 Fig). To have a more balanced representation, we centre category weights with their world values. We also weight the categories with their contribution *C*_*i*_ instead of their average proportion *q*_*i*_, because this choice does not result in visible changes on the maps (except that the size of the Neuroscience category is smaller with *C*_*i*_ as this point has a central position in the cloud of category points) and because it is consistent with the value of the overall interdisciplinary index. With this representation, the value of the overall index is exactly the difference between the sum of surfaces of positive contributions (red bubbles on the figure) and the sum of surfaces of negative contributions (blue bubbles). Fig 4 shows increasing total weight of red points and decreasing total weight of blue points when shifting from Aix-Marseille to Strasbourg and to Paris 7 universities. It also shows different profiles of references for the three universities with a focus on basic biology in Strasbourg, on psychology, sport sciences and physiology in Aix-Marseille and with a large range of categories ranging from psychology, to medical research and basic biology in Paris 7 University.

**Table 4:** Contributions of categories to the overall interdisciplinarity indicator for Aix-Marseille, Strasbourg and Paris 7 universities.

	Category	Ci	Z-score
**AIX-MARSEILLE**			
VX	PSYCHOLOGY, EXPERIMENTAL	1.053	8.915
UM	PHYSIOLOGY	0.826	8.358
XW	SPORT SCIENCES	0.479	4.434
VI	PSYCHOLOGY	0.300	4.980
OT	LINGUISTICS	0.236	4.325
VE	*PSYCHIATRY*	-0.239	1.318
DQ	CARDIAC & CARDIOVASCULAR SYSTEMS	-0.281	-16.960
MA	HEMATOLOGY	-0.285	-11.895
BA	ANESTHESIOLOGY	-0.290	-7.073
DR	CELL BIOLOGY	-0.324	-3.669
PY	MEDICINE, GENERAL & INTERNAL	-0.378	-8.894
NI	IMMUNOLOGY	-0.462	-10.426
YA	SURGERY	-0.484	-6.897
ZD	PERIPHERAL VASCULAR DISEASE	-0.553	-15.999
IA	ENDOCRINOLOGY & METABOLISM	-0.587	-6.715
TU	PHARMACOLOGY PHARMACY	-0.906	-11.372
CQ	BIOCHEMISTRY & MOLECULAR BIOLOGY	-1.040	-8.692
RT	CLINICAL NEUROLOGY	-1.137	-7.840
**STRASBOURG**			
CQ	BIOCHEMISTRY & MOLECULAR BIOLOGY	1.062	5.067
KM	GENETICS & HEREDITY	1.004	4.945
TU	PHARMACOLOGY & PHARMACY	0.935	5.275
DR	CELL BIOLOGY	0.553	3.544
IA	*ENDOCRINOLOGY & METABOLISM*	0.365	2.086
VE	*PSYCHIATRY*	0.323	1.505
HY	*DEVELOPMENTAL BIOLOGY*	0.260	2.238
RU	*NEUROSCIENCES*	0.233	1.381
KI	GASTROENTEROLOGY & HEPATOLOGY	-0.280	-20.395
TC	ORTHOPEDICS	-0.285	-16.436
EP	COMPUTER SCIENCE, ARTIFICIAL INTEL	-0.286	-7.824
WC	REHABILITATION	-0.304	-22.906
TD	OTORHINOLARYNGOLOGY	-0.316	-42.029
NI	IMMUNOLOGY	-0.320	-3.454
XW	SPORT SCIENCES	-0.365	-2.930
PY	MEDICINE, GENERAL & INTERNAL	-0.375	-5.553
RX	NEUROIMAGING	-0.376	-7.101
ZD	PERIPHERAL VASCULAR DISEASE	-0.402	-4.750
VX	PSYCHOLOGY, EXPERIMENTAL	-0.506	-5.000
YA	SURGERY	-0.560	-4.520
VY	RADIOLOGY, NUCLEAR MED & MED IMAGING	-0.571	-3.667
RT	CLINICAL NEUROLOGY	-0.716	-2.616
**PARIS 7**			
RT	CLINICAL NEUROLOGY	1.370	4.799
KM	GENETICS & HEREDITY	0.878	4.620
KI	*GASTROENTEROLOGY & HEPATOLOGY*	0.674	2.540
TD	OTORHINOLARYNGOLOGY	0.626	2.715
TQ	PEDIATRICS	0.604	5.089
DM	ONCOLOGY	0.545	2.954
TM	PATHOLOGY	0.388	3.820
VE	*PSYCHIATRY*	0.331	1.332
CQ	*BIOCHEMISTRY & MOLECULAR BIOLOGY*	0.280	1.319
HY	DEVELOPMENTAL BIOLOGY	0.275	2.655
ZD	*PERIPHERAL VASCULAR DISEASE*	0.264	1.918
AA	*ACOUSTICS*	0.202	1.418
VJ	PSYCHOLOGY, MULTIDISCIPLINARY	-0.209	-5.619
XW	SPORT SCIENCES	-0.224	-2.854
IA	*ENDOCRINOLOGY & METABOLISM*	-0.232	-2.203
RX	NEUROIMAGING	-0.285	-4.996
VI	PSYCHOLOGY	-0.326	-6.782
TU	*PHARMACOLOGY & PHARMACY*	-0.346	-2.499
CN	BEHAVIORAL SCIENCES	-0.570	-11.948
VX	PSYCHOLOGY, EXPERIMENTAL	-0.769	-10.308

Contributions are sorted with decreasing values and displayed if their absolute value*100 is larger than 0.2. Non significant scores at 1% level are *italicized*. Complete data to be found on the GitHub repository

In order to check the interpretation of category contributions, we collected the exploitable information on laboratory addresses retrieved from the *address* field in the WoS database. In about half of the addresses, the laboratory name was present and could be disambiguated, despite the possible variation of units contours and names over the period (Table 5). Unit names provide information on their research themes and focus, confirmed by information from the web sites, which can be related with categories of most cited references.

**Table 5:** Number of publications with identified research units for Aix-Marseille, Strasbourg and Paris 7 Universities.

Unit code	Acronym	Unit name	number	%
**AIX-MARSEILLE**				
		CNRS-AIX MARSEILLE University joint units		**61%**
FR 3512	FR 3C	Federation 3C Comportement Cerveau Cognition	303	34%
UMR 7291	LNC	Lab Neurosciences cognitives (former UMR 6155)		
UMR 7260/6149	NIA	Lab Neurosciences Integratives & Adaptatives		
UMR 7290	LPC	Lab Psychologie Cognitive		
UMR 6193	INCM	Mediterranean Institute for Cognitive Neuroscience		
UMR 6216	IBDL	Developmental Biology Institute of Marseille	70	8%
UMR 7287	ISM	The Institute of Movement Sciences	57	6%
UMR 6196	P3M	Plasticity et Physiopathology of Motricity	37	4%
UMR 6231/7286	CRN2M	Center for Research in Neurobiology and Neurophysiology of Marseille	72	8%
		INSERM-AIX MARSEILLE University joint units		**24%**
FHU	Epinext	Epilepsy and Disorders of Neuronal Excitability	145	16%
U 901	INMED	Institut de Neurobiologie de la Méditerranée		
U 1106	INS	Institut de Neurosciences des Systèmes		
U 751		Epilepsies & Cognition (Hopital La Timone)	66	7%
		Other disambiguated addresses	137	15%
		Total number of disambiguated addresses (54.2% of total)	887	100%
**STRASBOURG**				
		CNRS-STRASBOURG University joint units		**83%**
UMR 7104	IGBMC	Institute of Genetics and Molecular and Cellular Biology	119	29%
	ICS	Mouse Clinical Institute (IGBMC facility)	7	2%
UPR 3212	INCI	Institute of Cellular and Integrative Neurosciences	114	28%
UMR 7191	LINC	Imaging and Cognitive Neurosciences Laboratory	95	23%
UMR 7178	IPHC	The Hubert Curien Pluridisciplinary Institute	8	2%
		INSERM-STRASBOURG University joint units		**16%**
U 666		Pathophysiology and cognitive pathopsychology of schizophrenia	43	10%
U 692		Central and Peripheral Mechanisms of Neurodegeneration	23	6%
		Other disambiguated addresses	4	1%
		Total number of disambiguated addresses (58.7% of total)	413	100%
**PARIS 7**				
		CNRS-PARIS 7 University joint units		**42%**
UMR 7592		Institut Jacques Monod	31	12%
UMR 8118		Brain physiology Lab (with Paris 6)	27	10%
UMR 8194	CESEM	Centre d’étude de la sensorimotricité (with Paris 5)	41	15%
UMR 8550	LPS-ENS	Physical Statistics	13	5%
		INSERM-PARIS 7 University joint units		**37%**
U 867		Minimal Invasive and Robotized Otological Surgery	6	2%
U 676	PROTECT	Promoting Research Oriented Towards Early Cns Therapies	77	29%
U 894	CPN	Psychiatry & Neurosciences Centre (Saint Anne Hospital)	15	6%
		CNRS-INSERM-PARIS 7 University joint unit		
UMR 8246/U 705		Neuropsychopharmacology and Drug Addictions Unit (with Paris 5)	30	**11%**
		Other disambiguated addresses	56	21%
		Total number of disambiguated addresses (32.2% of total)	266	100%

Aix-Marseille neuroscientists cite much less articles in medical research and biology than the world average behaviour and they favour references in psychology, sport sciences and physiology. This overall orientation of neuroscience research in Aix-Marseille results from the weight of CNRS joint research units focused on fundamental research in cognitive psychology and integrative neuroscience as the Federation 3C and the Mediterranean Institute for Cognitive Neuroscience (34% of disambiguated addresses, see Table 5). References in sport science and physiology are to be related with the publications of the Institute of Movement Sciences and of the Plasticity and Physiopathology of Motricity Unit (11% of disambiguated addresses). Medical research is achieved mainly in INSERM joint units (24% of disambiguated addresses), some of them organised in a recently labelled Hospital-University Research Department (DHU) on brain pathologies (EPINEXT Federation—Epilepsy and Disorders of Neuronal Excitability). Beyond the quality of research, more interaction between these different orientations is wished by the university management. New facilities have been recently installed as the Institut de Neurosciences de La Timone, in order to favour more efficient collaboration among the different skills to “bridge the gap between fundamental and clinical research and develop an integrative approach aiming at designing new therapeutic strategies and care management processes” (see http://www.int.univ-amu.fr/?lang=e). This confirms that the lack of integration captured by the indicator is a subject of attention from managers.

References in Strasbourg publications have a very different profile centred on biochemistry, cell biology, genetics and pharmacology. Similarly to Aix-Marseille, references in medical disciplines are low, compared to world average. This fundamental biology orientation of neuroscience research is to be related with the weight of the production of two research institutes: the Institute of Cellular and Integrative Neuroscience (INCI-CNRS) with research programmes that “aim at understanding the development and function of the nervous and neuroendocrine systems at the molecular, cellular and integrative levels”, and the Institute of Genetics and Molecular and Cellular Biology (IGBMC) which domains of investigation range from developmental biology to integrative structural biology, via functional genomics, cancer, translational medicine and neurogenetics. These two institutes contribute for 28% and 30% of the addresses of our sample. The Imaging and Cognitive Neurosciences Laboratory (LINC)—now Laboratoire de Neurosciences Cognitives et Adaptatives (LNCA)—with 23% of addresses in our sample is focused on the study of cognitive function and nervous system pathologies. Only two INSERM research units located at the Strasbourg Hospital were identified among the disambiguated lab addresses (U66 and U692, see Table 5). The relative weights of the research units in our sample is thus consistent with the references profile provided by category contributions.

Unlike Aix-Marseille and Strasbourg, Paris 7 references exhibit a wide range of disciplines involving basic research in biology as well as medical research. For instance, the link with Clinical Neurology is stronger than the world average, as those with Pediatrics, Otorhinolaryngology, Oncology and Pathology. Among the identified unit addresses, about a half are from medical research units with INSERM with research themes as early central nervous system therapies (PROTECT unit), psychiatry (Psychiatry and Neuroscience Centre at Sainte Anne Hospital) or Neuropharmacology and Drug Addiction (see Table 5). The CESEM unit—Centre d’études de la sensori-motricité—has a large scope of research themes and is particularly involved in health care delivery and education, feature underlined by the 2009 HCERES evaluation of the unit. The high value of the *within* index of Paris 7 University suggests that integration is remarkable at the publication level and it is interesting to note that the share between medical and basic biology research is well balanced, including a research unit associated with both CNRS and INSERM.

**Fig 5:**
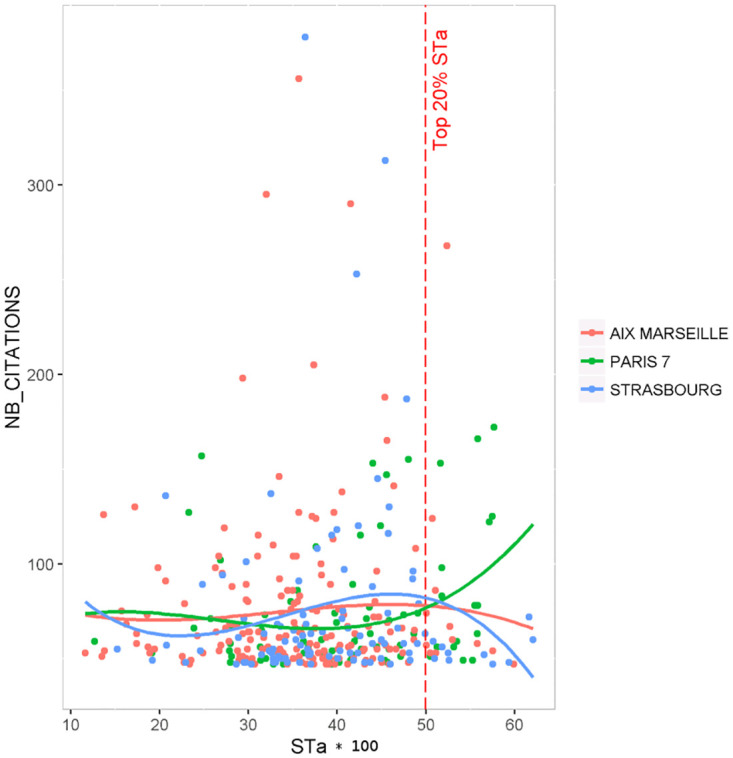
Article interdisciplinarity index *STa* and number of citations in a 3 year window for Aix-Marseille, Strasbourg and Paris 7 Universities. Displayed articles are in the top 20% percentile of the most cited publications. A polynomial curve with degree 3 is fitted for each university.

Considering again the relationship between interdisciplinarity and citation number, but now at the article level, and zooming on the papers in the top 20% percentile of most cited papers, we observe that the three universities have different distribution of number of citations versus interdisciplinarity (Fig 5). The proportion of articles in the top 20% percentile of most interdisciplinary papers (i.e. greater than 0.50) are 5.3% for Aix-Marseille, 10.4% for Strasbourg, and 21.2% for Paris 7. For this latter university, the proportion is slightly higher that the 20% expected if the number of citation and interdisciplinarity index were independent variables. This shows that it possible to locally reduce the curse of highly interdisciplinary papers not to be the most cited ones.

The cases of Aix-Marseille and Strasbourg, where a low integration between fundamental and medical research is observed, reflects a specific feature of the organisation of French public research. Collaborations between national research institutions on one hand and universities on the other hand is often organized through common labs (i.e. Unités Mixtes de Recherche). In these two universities, common labs are established either with CNRS or with INSERM with a higher number with CNRS. The different missions of the two institutions could partially explain the low integration observed in these two universities. This hypothetical explanation would require further investigation but it seems to be shared by research managers who incite to merge units into larger structures involving more research institutions and bridging the gap between hospital and basic research.

### Synthetic comparison of eight universities

**Fig 6:**
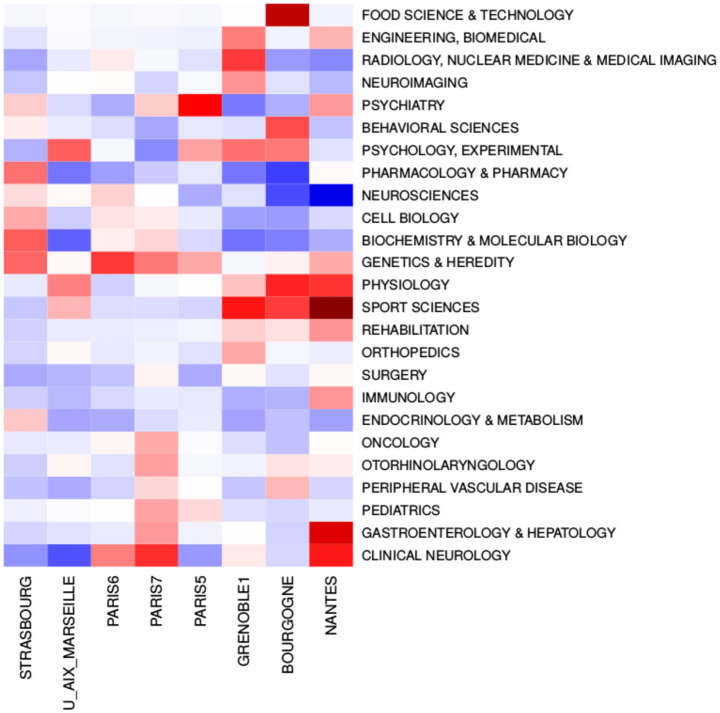
Comparison of category contributions for 8 universities. Red cells correspond to positive values of the centred contributions, while blue cells correspond to negative values. White cells correspond to contributions with p-values larger than 0.1. Categories are displayed only if the centred contribution is greater than 0.5 or smaller than -0.5 for at least one of the eight universities. Data in *data_integer/stat_results* files in https://github.com/turckheim/interdisciplinarity.

Identifying laboratories from addresses registered in the WoS database is not easy but it aimed at checking that the profile of references of a university is correctly related with the labs research themes. Universities that have publication lists by laboratory could refine such an analysis. We do not further develop this approach with the other universities of this study. When the aim is to position and compare universities and provide evidence for an overall strategic reflection, a more synthetic representation is useful as achieved with a heatmap. In Fig 6, the values of contributions are represented on a colour scale from dark blue for the lowest contribution to dark red for the highest. This figure shows for example that Paris 5 has a very different profile from that of Paris 7, despite the number of their joint publications. The three universities of Grenoble 1, Bourgogne and Nantes mainly cite publications in sport science, physiology, and experimental psychology journals. The category profiles confirm some specialities of these universities as imaging techniques in Grenoble or food science and technology in Bourgogne, related to a long term regional strategy to develop innovation through interactive research with food industries as in the joint Centre des sciences du goût et de l’alimentation (https://www2.dijon.inra.fr/csga/index_eng.php). Two units on specific research themes in Nantes are focused on the digestive nervous system (Neuro-gastroenterology—Inserm Unit U 913) and on physiology, mechanics, psychology and cognitive ergonomics (Laboratoire Motricités, Interactions, Performances—MIP) that explains the high contribution of Gastroenterology, of Sport Sciences and of Physiology for this university. Moreover, other units publishing in neuroscience in Nantes, representing about half of disambiguated addresses, are medical research units, not specialised in neurology. In these units, neuroscience therefore represents a complementary approach with an accordingly interdisciplinary approach, possibly explaining the high values of the Nantes interdisciplinarity indexes.

This analysis and the discussion with university representatives in the working group confirmed that, in this study, the information provided by the indicators was interpretable and consistent with nearby universities information. Therefore it is acceptable to consider the quantitative results provided by these indicators as a tool for introducing panel work on scientific policies aiming at sharing experience on how to incite and to manage interdisciplinary research.

## Discussion

This work is based on various assumptions and technical choices that are questionable.

Reducing the measure of interdisciplinarity to the diversity of references in publications is a first short cut. References do not say anything about the process and the dynamics which led to interdisciplinary output. Information about the disciplinary orientation of authors co-signing publications can be used to track interdisciplinary collaborations. These disciplinary orientation of individual researchers can be inferred from their previous publications [12] or from the scientific departments they are affiliated [11]. The history of research themes of the teams or of individual researchers contains relevant information for understanding the cognitive dynamics happening in such processes and for comparing it with the synthesised information provided by bibliometric indicators of output [22]. Such an approach requires the collection of historical data and interview based information.

An alternative source of information in documents is in title and abstract or in whole text of articles. For example, topics as produced by fitting a topic model [23, 24] on whole texts or on summaries of an overall corpus allow to describe each document as a combination of topics, each of them being related to a scientific discipline [25]. In complement to the measure of the diversity of sources, the range of disciplines impacted by publications is another aspect. A diffusion score based on the diversity of citing is usefully benchmarked with interdisciplinarity of sources or integration score [16, 26].

The choice of an integrated measure of diversity is a convenient one but it hides the different dimensions of diversity blending variety, balance and disparity in a specific way that may not be the optimal recipe as claimed by Zhang and co-authors [27]. Moreover, it prevents from distinguishing between the three dimensions of interdisciplinarity whereas these components measure different ways to integrate disciplines. For instance, it prevents from distinguishing between close (or proximal [6]) and remote (or distal) interdisciplinarity and deciphering the relationship of these different dimensions of interdisciplinarity with impact. The effect on short term citations of these different dimensions leads to contradictory results [6, 28, 29], partly due to different decompositions of interdisciplinarity. However, it is suggested that in a long term perspective (citations in a 13-year window), variety and disparity may have a positive effect on impact [28]. As remote interdisciplinarity is much more demanding from researchers and institutions, it may be worth to use some of these other indicators to analyse specific strategic options.

In this paper, we use a classification of scientific papers derived from the classification of journals into the WoS categories. A larger base of publications would be necessary to capture more references in social sciences and humanities, taking into account both WoS and Scopus databases, as the UCSD classification of journals into 554 sub-disciplines [30]. It is also possible to use a classification of articles that does not take journals into account but only citation links between publications. The CWTS classification [31] provides an alternative choice for categories and their 672 middle level clusters could also be used as a classification of references. However interpretable category contributions do not easily result from such a refined classification.

Field delineation is another important issue. As mentioned earlier, there is no consensus on the set of journals where Neuroscience research is published. Merging the two Wos Categories Neuroscience and Clinical Neurology—which share about 25% of articles—or choosing Neuroscience journals as defined in Scimago could lead to different results. This means that users and experts should decide how to select the journals or the articles to consider in such a study.

Finally, one has to be aware that a high interdisciplinary indicator cannot be a science policy objective in itself. First there are many possible indicators and second, highly interdisciplinary research is not equivalent to good science nor to societal effectiveness [6]. This is the hazard of any indicator to unduly replace political objectives and interdisciplinarity indicators do not escape this trap better than others. However, interdisciplinarity may be a technical milestone of institutions management strategy as it is a step towards opening research options and facilitating interaction with stakeholders and therefore favour various types of societal impact [32].

## Conclusion

Based on the Rao-Stirling diversity index, this work proposes a new way to scrutinize interdisciplinary practices. The two components of the overall diversity of sources of the publications of an institution can be interpreted as proxies for the interdisciplinary integrated component of this diversity and its not yet integrated or multidisciplinary component. The decomposition of the overall index as a profile of disciplines meets the demand of users to look *inside* the overall indicator and to have meaningful and easily interpretable graphical representations. These two decompositions provide institutions with a flexible tool to explore the diversity of disciplines in publication references for each research field. The application of this tool to neuroscience research in French universities shows that it is appropriate to reveal a diversity of practice in terms of interdisciplinarity in a field. These practices could result from an explicit strategy, supported by incentives measures or just emerge in a given institutional setting. In both cases, it is necessary to confirm the interpretation of the quantitative observations provided by these indicators, and further explore the practises to understand which context or policy measures could explain high or low values of the indicators. Applied to all French universities involved in neuroscience research, these indicators make it possible to describe the landscape of interdisciplinary orientation of French neuroscience at the institution level. These indicators could also be used at different levels, as for positioning countries in a global landscape. As other science and technology indicators, these indicators could be successfully used for debating between organisations sharing a common research domain.

## Material and methods

Data consist of documents of the four types *article, letter, note* and *review* published over the period 2008–2014 in journals classified in the *Neurosciences* category in the Thomson Reuters database at OST, updated in 2013 with TR updates and with the affiliation validation process carried out by OST with French universities. Documents with less than three references in the WoS are not taken into account.

For the overall index which is based on the references of the whole corpus, the references of each document are weighted by the inverse of the number of references of the document, so that the set of references for each document has the same weight. This option, called EWA (Equal weight by article) in [8] is recommended because it makes it possible to include both review papers and articles.

For a reference in a journal assigned to many categories, a reference was counted in each category (whole counting option). The fractional counting option was also tested but no important difference with the whole counts occurred in the results. We do not use the “Multidisciplinary” WoS category and articles in these journals are classified in another category on the basis of their references.

Similarities between categories have been calculated as [18] by the mean of a matrix of citation flows between WoS categories, and then converted it into a Salton’s cosine similarity matrix in the citing dimension. The similarity in the citing patterns for each pair of WoS categories have been calculated for period 2008–2012, for 248 WoS categories. The VosViewer map displaying a 2D representation of category similarities is available at S2 Fig.

To visualise the statistical precision of the indicators, it is useful to show their confidence intervals for a chosen probability level. As the two components *ST*_*W*_ and *ST*_*B*_ of *ST* are not independent random variables, a confidence area with a given probability is an ellipsis, which axis orientation depends on the correlation of the two components [33]. In S3 Fig, we show the confidence areas of probability 0.98 for the two-dimensional indicator (*ST*_*W*_, *ST*_*B*_) for the eight universities of this study.

Data and scripts availability. Reference counts by category for the publications of the eight universities (for full or fractional counting method) and for two fields Neuroscience (RU) and Clinical Neurology (RT) are available at https://github.com/turckheim/interdisciplinarity in *data_integer* or *data_frac* folders. Basic data (i.e. list of references for each publication) are provided under licence by Thomson Reuteurs (now Clarivate Analytics) and are available with an access to Web of Science Core Collection. Results for the whole database 2008–2012 namely i) the category similarity matrix, ii) world statistics *ST*, *ST*_*W*_, *ST*_*B*_ as well as the percentiles of the article index world distribution, and all statistical results for the 8 universities used in the tables and figures of this paper are given in the above GitHub repository.

R scripts for computing the values of the indicators and their statistics are as well available in the *R_scripts* folder and commented in the *Steps.pdf* file.

## Supporting Information

S1 Appendix Statistical properties of Rao-Stirling indicators of interdisciplinarity. (PDF)

S1 Fig Within and between indicators of French institutions for seven fields. Astronomy and Astrophysics (BU), Behavioral Sciences (CN), Biochemical Research Methods (CO), Environmental Sciences (JA), Clinical Neurology (RT), Neurosciences (RU), Pharmacology & Pharmacy (TU).
(PDF)

S1 Table Number of citations and interdisciplinarity indexes for the publications of the study. Three year citation window.
(ZIP)

S2 Fig VOSviewer base map of WoS categories based on Salton’s cosine similarities in the citing dimension. The networkfile is *category similarity matrix.txt* on the GitHub repository. Cluster resolution is 1.5 which provides 6 clusters.
(PDF)

S2 Table VOSviewer input files for Fig 4 and S2 Fig. (ZIP)

S3 Fig Confidence ellipses for the pairs (*ST*_*W*_, *ST*_*B*_) with probability 0.98. If an ellipsis does not cross the second bisector (i.e. the line *x* + *y* = 0), the overall indicator *ST* = *ST*_*W*_ + *ST*_*B*_ is significantly different from zero at level 0.02. If the ellipses of two universities do not overlap, their pairs (*ST*_*W*_, *ST*_*B*_) are significantly different at level 0.04.
(TIFF)
