# Supplementary material for: Analysing Institutions Interdisciplinarity by Extensive Use of Rao-Stirling Diversity Index
Source: PLoS One. 2017 Jan 23;12(1):e0170296. doi: 10.1371/journal.pone.0170296 (PMC5256946; doi:10.1371/journal.pone.0170296)
Supplement: S1 Appendix — (PDF) [file pone.0170296.s001.pdf]

Theoretical support for the R programs of the project
Interdisciplinarity indicators for research institutions
CC-BY licensed document

Statistical properties of Rao-Stirling indicators of interdisciplinarity

Lorenzo Cassi · Wilfriedo Mescheba · Élisabeth de Turckheim

August 2015

Introduction: using the statistical properties of bibliometric indicators

One methodological issue with bibliometric indicators is to define a lower bound for the size of the corpus where an indicator is computed to insure that the value of the indicator is statistically *significant*. The aim is to prevent abusive interpretation when the value of the indicator could be too much affected by errors or exceptional values in the data.

If a probabilistic model is defined and the indicator considered as a random variable in this model, the statistical properties of this variable provide some answer to this question. The basic idea is that an indicator provides relevant information if its value is different from a reference value and if this difference is large enough with respect to its variability. If the indicator is the average of a measure over the n documents of a corpus, or if it is a function of such averages, the variance of the indicator decreases at the rate $1/n$. Therefore, thanks to a central limit theorem, the p-value of the indicator - which is the probability that the difference of the indicator with a reference value is at least larger than the observed difference - is available.

In this paper, we show how to compute the variance of the Rao-Stirling interdisciplinarity estimator (Stirling, 2007; Rafols and Meyer, 2010), of its *within* and *between* components defined in Cassi et al. (2014) and of its cat-

L. Cassi · W. Mescheba · E. de Turckheim
HCERES-OST - Observatoire des Sciences et des Techniques, 75002 Paris, France
E-mail: (lorenzo.cassi, wilfriedo.mescheba, elisabeth.deturckheim)@obs-ost.fr

L. Cassi
Université Paris 1, Centre d'économie de la Sorbonne, 75013, Paris, France
Address for correspondence: lorenzo.cassi@univ-paris1.fr

E. de Turckheim
INRA, Délégation à l'évaluation, 75007 Paris, France
E-mail: elisabeth.deturckheim@paris.inra.fr

egory components used in Cassi et al. (2015). For each indicator, a z-score, which is a standardized normal variable - similar to the Student's t statistic - provides the associated p-value.

Acknowledgement

This document is mainly extracted from Appendix B of Cassi et al. (2014) with the following modifications:

- the notations have been improved, hoping that they will be more readable for a non statistician reader,
- since it is the choice recommended in the mentioned article, only the case with equal weights on citing articles - the EWA case - is presented here,
- the variance of the category contributions which were not used in Cassi et al. (2014) has been added, also derived from the delta method theorem,
- different choices for the graphical representation of the interdisciplinary indicators are discussed that take into account the probabilistic information associated with each indicator.

1 Probabilistic model

In order to derive statistical properties of the indicators, we choose a simple model for the distributions of the references into categories. In this model, the observed variables are the counts of references of each article a in each category i, denoted N_{ai}. The model is a two-step random model. For each article a of the n articles of the corpus, the first step selects random values for

- the number N_a of its references,
- the (non observed) true value p_a of the proportions of its references in each of the m categories

$$p_a = (p_{a1}, \dots, p_{ai}, \dots, p_{am}).$$

These n variables (N_a, p_a) are supposed independent and identically distributed (i.i.d.) with mean value (N^0, p^0)

$$p^0 = (p^0_1, \dots, p^0_i, \dots, p^0_m).$$

The second step selects for each article a the number of references in the m categories $(N_{a1}, \dots, N_{ai}, \dots, N_{am})$ as a multinomial distribution with parameters N_a and p_a. The global interdisciplinarity indicator is computed with the average values q_i of N_{ai}/N_a

$$q_i = \frac{1}{n}\sum_{a=1}^{n}\frac{N_{ai}}{N_a}$$

$$ST = \sum_{i,j} q_i q_j d_{ij}$$

where the 'distances' d_{ij} are fixed known parameters[1]. This model ensures that the conditional mean of N_{ai}/N_a given (N_a, p_a) is p_{ai} and its (unconditional) mean is p_i^0

$$E(N_{ai}/N_a) = E(q_i) = p_i^0.$$

Such a model allows to approximate the distribution of the empirical index ST with a normal distribution with mean ST^0

$$ST^0 = \sum_{i,j} p_i^0 p_j^0 d_{ij}$$

and a variance which can be estimated.

2 A general central limit theorem

For statistical inference based on the empirical value of an index, we need to compute the distribution of variables such as

$$\sqrt{n}\left(ST - ST^0\right).$$

This distribution is available for large samples when a central limit theorem holds. When $\hat{\sigma}$ is a convergent estimation of the variance σ of ST, the normalized statistic or z-score denoted z^*

$$z^* = \frac{\sqrt{n}}{\hat{\sigma}}\left(ST - ST^*\right) \tag{1}$$

can be used to compare the true (or theoretical) value ST^0 to a reference value ST^*.

The useful theorem is derived from a central limit theorem for a smooth function f of averages of i.i.d. random variables. This theorem is based on the delta-method which uses the first order Taylor expansion of the function f. This theorem is available with different formulations in various teaching documents as the Lecture Notes in Statistics by Geyer (2001, theorem 8.9, page 221). We formulate the theorem with convenient notations for our situation.

Theorem 1 (Delta method) *Let $(X_{1a}, X_{2a}, \ldots, X_{ma})$, $a = 1, 2, \ldots, n$, denote an i.i.d. n-sample from a m-dimensional distribution with mean $(E(X_1), E(X_2), \ldots, E(X_m))$ and consider a function $f = f(X_1, X_2, \ldots, X_m)$ of the averages*

$$X_i = \frac{1}{n}\sum_{a=1}^{n} X_{ia}.$$

Then, for f sufficiently smooth, the variables

$$W_n = \sqrt{n}\left(f(X_1, X_2, \ldots, X_m) - f(E(X_1), E(X_2), \ldots, E(X_m)\right)$$
$$W_n^1 = \sqrt{n}\sum_{i=1}^{m} \lambda_i (X_i - E(X_i))$$

[1] As shown in Cassi et al. (2014), the decomposition into the *within* and *between* components holds if d_{ij} is the square of a Euclidean distance.

have the same asymptotic distribution which is normal with mean zero and variance σ_F^2, where the coefficient λ_i is the partial derivative of f at $(E(X_1), E(X_2), \ldots, E(X_m))$

$$\lambda_i = \frac{\partial f}{\partial x_i}(E(X_1), E(X_2), \ldots, E(X_m))$$

and σ_F^2 is the variance of $F_a = \sum_i \lambda_i X_{ia}$.

The theorem follows from the fact that, when the second order derivatives of f are bounded, the function $f(X_1, \ldots, X_n) - f(E(X_1), E(X_2), \ldots, E(X_m))$ and its first order expansion $\sum_i \lambda_i (X_i - E(X_i))$ have the same asymptotic distribution.

3 Asymptotic distribution of the global index

A straightforward use of this general theorem with $X_{ia} = N_{ai}/N_a$, $X_i = q_i$, $E(X_i) = p_i^0$ and with the function h

$$h((x_i)_{i=1,\ldots,m}) = \sum_{i,j} d_{ij} x_i x_j$$

provides the asymptotic distribution of

$$\sqrt{n}\left(ST - ST^0\right) = \sqrt{n}\left(h((X_i)_i) - h((E(X_i)_i)\right).$$

As the the partial derivatives of h at $(E(X_1), E(X_2), \ldots, E(X_m))$ are

$$\lambda_i = \sum_j (d_{ij} + d_{ji}) p_j^0 = 2 \sum_j d_{ij} p_j^0 = 2\gamma_i^0$$

applying Theorem 1 provides the asymptotic distribution of $\sqrt{n}\left(ST - ST^0\right)$.

Corollary 1 *The global interdisciplinarity index ST is such that the distribution of*

$$\sqrt{n}(ST - ST^0) = \sqrt{n} \sum_{i,j=1}^{m} \left(q_i q_j - p_i^0 p_j^0\right) d_{ij}$$

can be approached with a centred normal distribution with variance σ_H^2 where σ_H^2 is the variance of

$$H_a = 2 \sum_i \gamma_i^0 \frac{N_{ai}}{N_a} \tag{2}$$

and $\gamma_i^0 = \sum_j d_{ij} p_j^0$.

To get the associated z-score as in (1) , we estimate γ_i^0 with $\gamma_i = \sum_j d_{ij} q_j$ and σ_H^2 with the empirical variance $\widehat{\sigma_H^2}$ of

$$\widehat{H_a} = \sum_i 2\gamma_i \frac{N_{ai}}{N_a}. \tag{3}$$

4 Asymptotic distribution of the *within* index ST^W

The within index $ST^W = \frac{1}{n}\sum_a ST_a$ is the average of article indexes ST_a where

$$ST_a = \sum_{i,j} \frac{N_{ai}}{N_a}\frac{N_{aj}}{N_a} d_{ij}$$

and its theoretical value ST^{W0} is the mean $E(ST_a)$ of ST_a. As the variables ST_a are i.i.d. variables, a standard central limit theorem is available for this indicator and ensures that

$$\sqrt{n}\left(ST^W - E(ST_a)\right) = \sqrt{n}\left(\frac{1}{n}\sum_a ST_a - E(ST_a)\right)$$

converges to a normal centred variable with variance equal to $\mathrm{Var}(ST_a)$. The usual Student's statistic is the corresponding z-score to compare the theoretical value ST^{W0} to a reference value ST^{W*}.

Corollary 2 *The* within *interdiciplinarity index ST^W is such that the distribution of*

$$\sqrt{n}(ST^W - ST^{W0})$$

can be approached with a normal distribution with mean zero and the same variance as ST_a.

5 Asymptotic distribution of the *between* index ST^B

As $ST^B = ST - ST^W$, this variable is a function of $(m+1)$ averages of independent variables which are the m variables X_i as above and $\frac{1}{n}\sum_a ST_a$. The first order Taylor expansion of $\sqrt{n}\left(ST^B - ST^{B0}\right)$ is just the difference of the first order expansions of $\sqrt{n}\left(ST - ST^0\right)$ and $\sqrt{n}\left(ST^W - ST^{W0}\right)$. Therefore the asymptotic distribution of $\sqrt{n}\left(ST^B - ST^{B0}\right)$ is straightforward.

Corollary 3 *The* between *interdiciplinarity index ST^B is such that the distribution of*

$$\sqrt{n}(ST^B - ST^{B0})$$

can be approached with a normal distribution with mean zero and variance σ_U^2, where σ_U^2 is the variance of U_a

$$U_a = H_a - ST_a$$

where H_a is as in (2).

To get the corresponding z-score, we estimate the variance σ_U^2 with the empirical variance of $\widehat{H_a} - ST_a$ and with $\widehat{H_a}$ as in (3).

6 Asymptotic variance of a category contribution to the global index

For a refined analysis, the global index ST can be split into the m contributions of individual categories according to

$$ST = \sum_i C_i,$$

where

$$C_i = q_i \sum_j q_j d_{ij} \,.$$

To evaluate the asymptotic variance of C_i we use Theorem 1 and we write $C_i = h^{(i)}((X_k)_{k=1,\dots,m})$ where $X_k = q_k$ and

$$h^{(i)}((x_k)_{k=1,\dots m}) = x_i \sum_j x_j d_{ij} \,.$$

As the partial derivatives of $h^{(i)}$ at $(E(X_1),\dots,E(X_m)) = (p_1^0,\dots,p_m^0)$ are

$$\begin{aligned} \lambda_k^{(i)} &= p_i^0 d_{ik} \quad \text{if} \quad k \neq i \\ \lambda_i^{(i)} &= \sum_j p_j^0 d_{ij} = \gamma_i^0 \end{aligned}$$

the asymptotic variance of $\sqrt{n}\left(C_i - C_i^0\right)$ is the same as the variance of

$$F_a^{(i)} = \sum_k p_i^0 d_{ik} \frac{N_{ak}}{N_a} + \gamma_i^0 \frac{N_{ai}}{N_a} \,. \tag{4}$$

Corollary 4 *The distribution of the contribution of category i to the global index is such that the distribution of $\sqrt{n}\left(C_i - C_i^0\right)$ can be approached with a normal distribution with mean zero and a variance σ_i^2 where σ_i^2 is the variance of $F_a^{(i)}$ as in (4).*

As before, to compute the associated z-score, we estimate the variance of $F_a^{(i)}$ with the empirical variance of $\widehat{F_a^{(i)}}$ where

$$\begin{aligned} \widehat{F_a^{(i)}} &= \sum_k q_i d_{ik} \frac{N_{ak}}{N_a} + \gamma_i \frac{N_{ai}}{N_a} \\ \gamma_i &= \sum_j q_j d_{ij} \,. \end{aligned}$$

7 Graphical representations of interdisciplinarity indicators

Choice of a corpus

To explore the interdisciplinary orientations of an institution, it is relevant to consider separately the publications of the different scientific departments or research programmes. The reason is that citation practices and sharing of knowledge with other disciplines is highly variable between scientific fields. Therefore, a global indicator computed with the whole set of publications of the institution will mainly depend on the balance between the different fields rather than on the specific interdisciplinary practices of the institution researchers.

Choice of a benchmark

When a specific research department of an institution is considered, a relevant benchmark is the corpus of the world publications in the same journals and in the same period of time. Therefore the difference of the indicators between the value for the institution and the world value - the *centred* indicator - should be displayed.

Representing z-scores or indicator values

The next point is to decide how to display both the value of the indicators and their degree of significance. Two choices are possible:

1. Display the z-scores associated with the centred estimators: as they have a normal distribution, z-score have a standard range of variation and their values can be easily interpreted in probabilistic terms: the greater the z-score, the more significant the value of the indicator. Shaded areas related to a chosen value of the p-value may be shown as an example. This choice was made in Cassi et al. (2014) (Fig. 1, Fig. 2). It is relevant when exploring the different research domains of an institution since a graph with the z-scores will show at once which research domains of the institution have an interdisciplinary orientation mostly different from the world standard.
2. Display the centred indicators: displaying z-scores is not adapted when comparing interdisciplinarity of a same scientific field in different institutions. The problem is that two institutions with a similar index will have very different scores if their sets of publications are very different in size. However, if both sets are large enough to ensure a reasonable level of significance, it is more informative to show the values of the indexes instead of the z-scores. In that case, two options are available to add information about the significance level
   - replace non significant values of the indicators by zero. This is the choice in Cassi et al. (2015) with a level equal to 0.01 (Fig. 3)

– display the value of the indicators as well as their confidence interval or confidence area associated to a chosen level (Templin, 2011, slides 15-16). An example for the 2-dimensional indicator (ST^W, ST^B) and the data of Neuroscience in eight French universities as in Cassi et al. (2015) is shown in Figure 4.

References

Cassi, L., Mescheba, W., and de Turckheim, É. (2014). How to evaluate the degree of interdisciplinarity of an institution? *Scientometrics*, 101(3):1871–1895.

Cassi, L., Mescheba, W., and de Turckheim, É. (2015). My interdisciplinarity *à moi*. An analysis of neuroscience research in French universities, 2008-2012. In *Research organizations under scrutinity. New indicators and analytical results*, pages 36–38. Universitá della Svizzera italiana, Lugano, ENID.

Geyer, C. (2001). *Stat 5102 Lecture notes.* University of Minnesota. http://www.stat.umn.edu/geyer/5101/notes/n2.pdf Accessed: 2013-08-21.

Rafols, I. and Meyer, M. (2010). Diversity and network coherence as indicators of interdisciplinarity: case studies in bionanoscience. *Scientometrics*, 82(2):263–287.

Stirling, A. (2007). A general framework for analysing diversity in science, technology and society. *Journal of The Royal Society Interface*, 4(15):707–719.

Templin, J. (2011). Multivariate normal distribution, Lecture 4, ICPSR Summer Session 2. http://jonathantemplin.com/files/multivariate/mv11icpsr/mv11icpsr_lecture04.pdf. Accessed: 2015-09-30.

Examples of figures

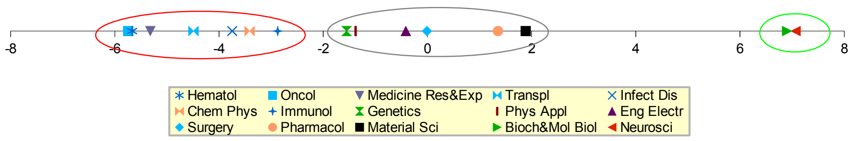

Fig. 1 Scores of the global centred indexes for 15 disciplines (WoS categories) for the university under study.Three groups of disciplines are identified with coloured ellipses. *Figure published in Cassi et al. (2014)*

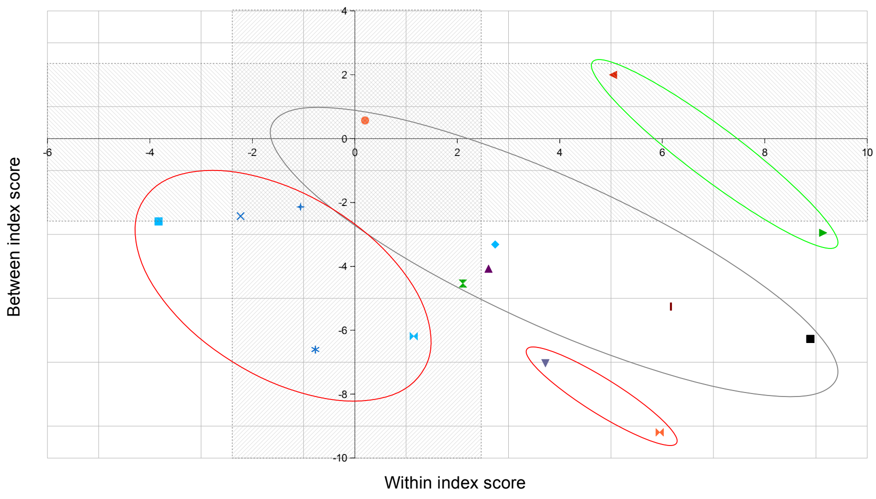

Fig. 2 Scores of the *within* and *between* centred indexes for 15 disciplines (WoS categories) of the university under study. Icons for categories are the same as in Fig 1 and the three groups of Fig. 1 are shown. Shaded areas correspond to scores which are not significant at level 1%. *Figure published in Cassi et al. (2014)*

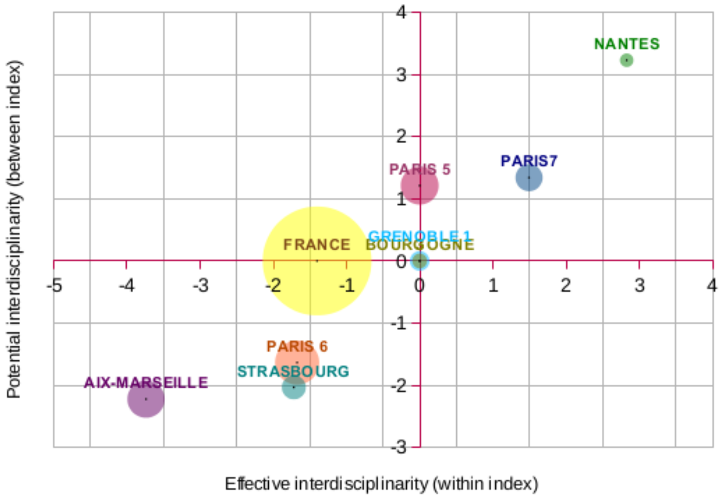

Fig. 3 Centred *within* and *between* interdisciplinarity indexes for neuroscience research in eight French universities. Non-significant values at level 1% have been replaced by zero. The surface of the bubbles is proportional to the number of publications. *Figure published in Cassi et al. (2015)*

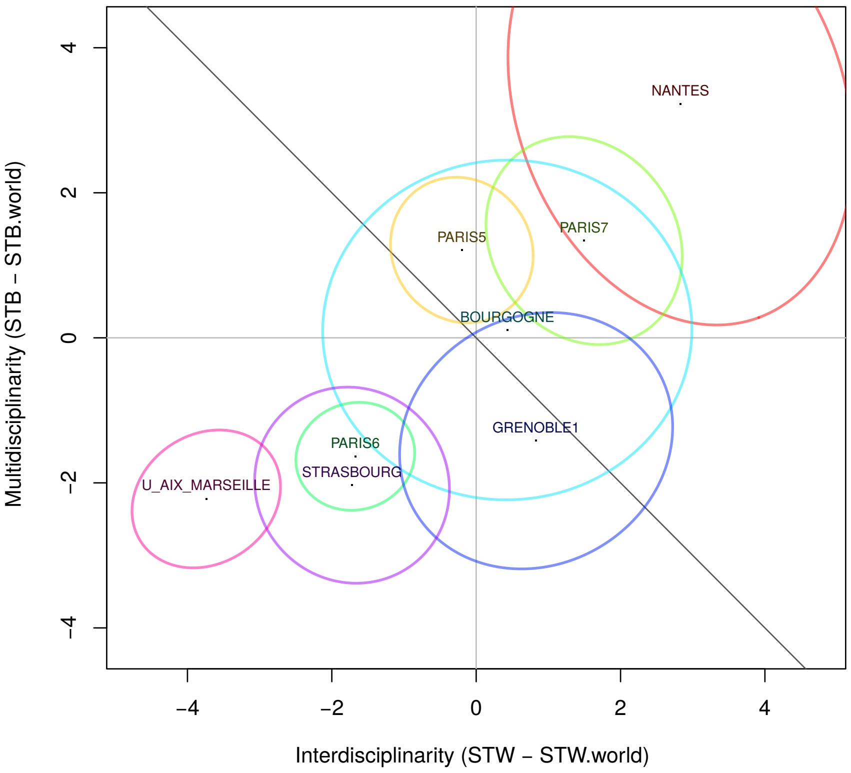

Fig. 4 Centred *within* and *between* interdisciplinarity indexes for neuroscience in eight French universities and their confidence areas of probability 0.98.
