# Supplementary material for: Analysing Institutions Interdisciplinarity by Extensive Use of Rao-Stirling Diversity Index
Source: PLoS One. 2017 Jan 23;12(1):e0170296. doi: 10.1371/journal.pone.0170296 (PMC5256946; doi:10.1371/journal.pone.0170296)
Supplement: S1 Fig — Astronomy and Astrophysics (BU), Behavioral Sciences (CN), Biochemical Research Methods (CO), Environmental Sciences (JA), Clinical Neurology (RT), Neurosciences (RU), Pharmacology & Pharmacy (TU). (PDF) [file pone.0170296.s002.pdf]

Within and Between Indexes of
French Research Institutions for seven Fields

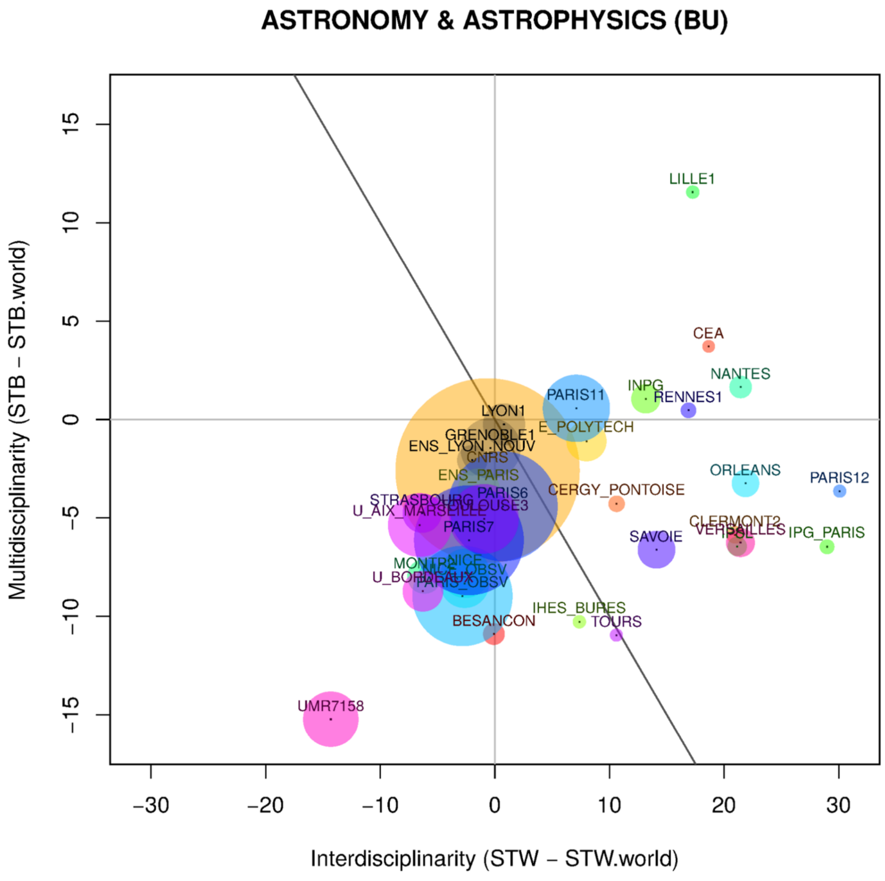

 S2 Fig

BEHAVIORAL SCIENCES (CN)

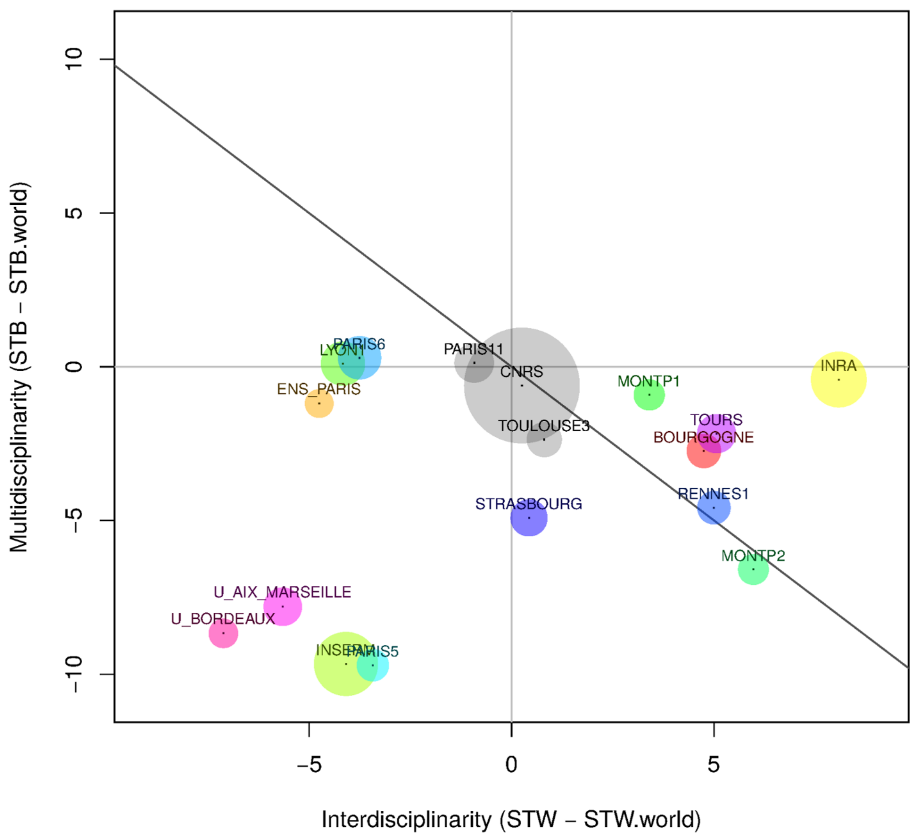

BIOCHEMICAL RESEARCH METHODS (CO)

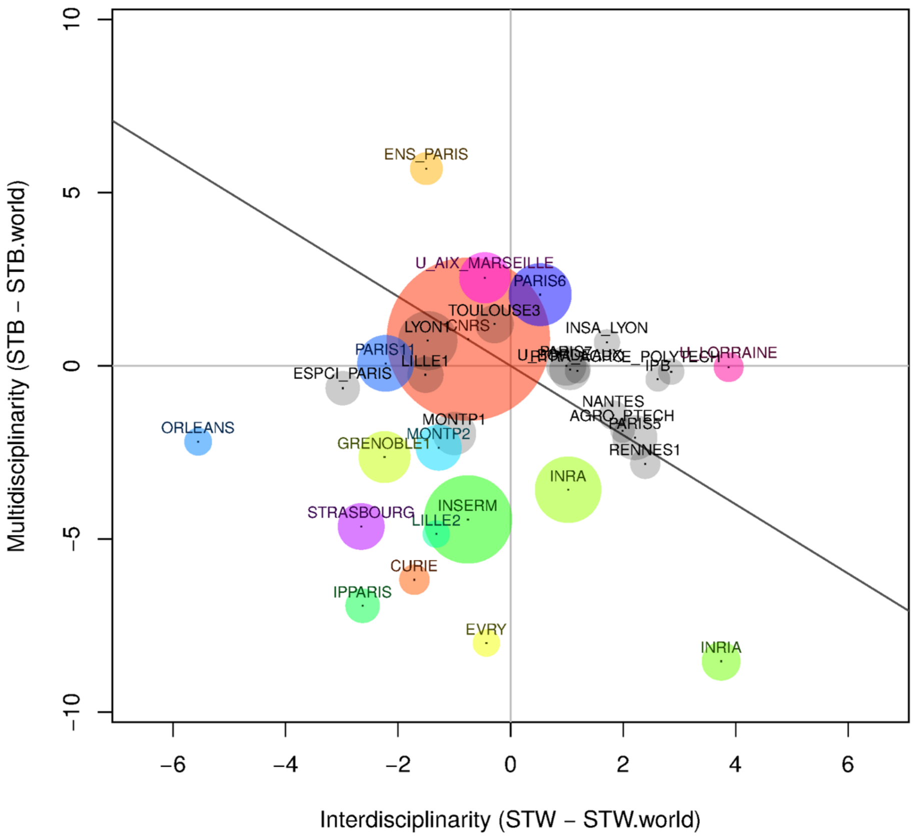

S2 Fig

ENVIRONMENTAL SCIENCES (JA)

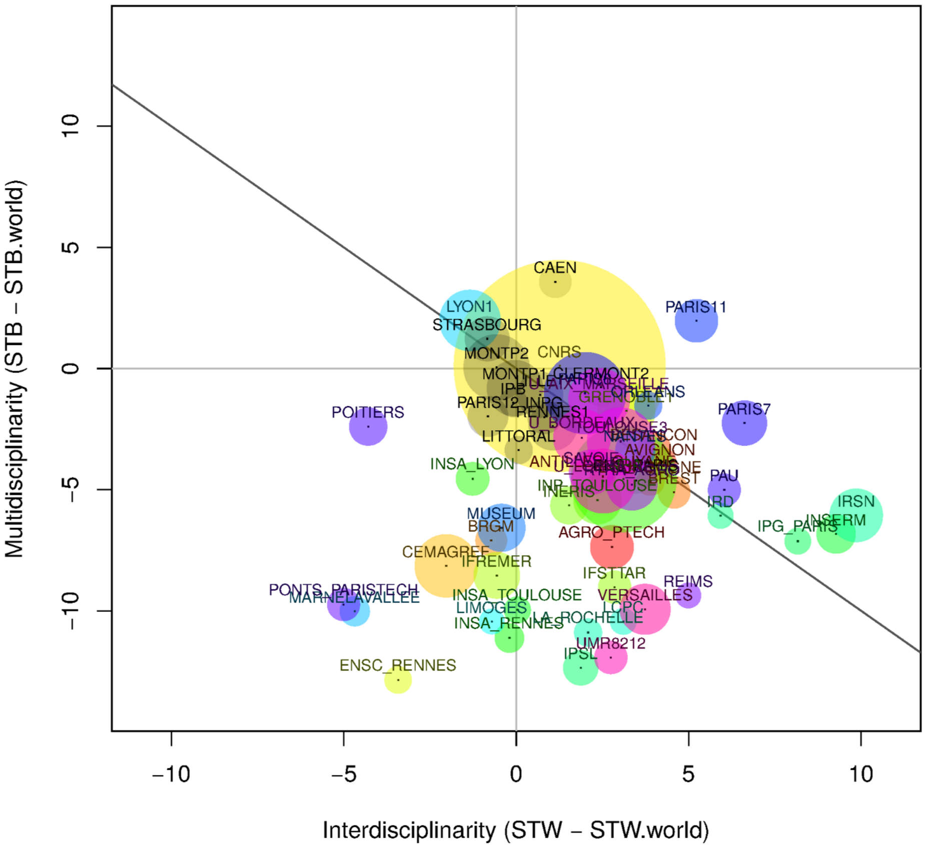

CLINICAL NEUROLOGY (RT)

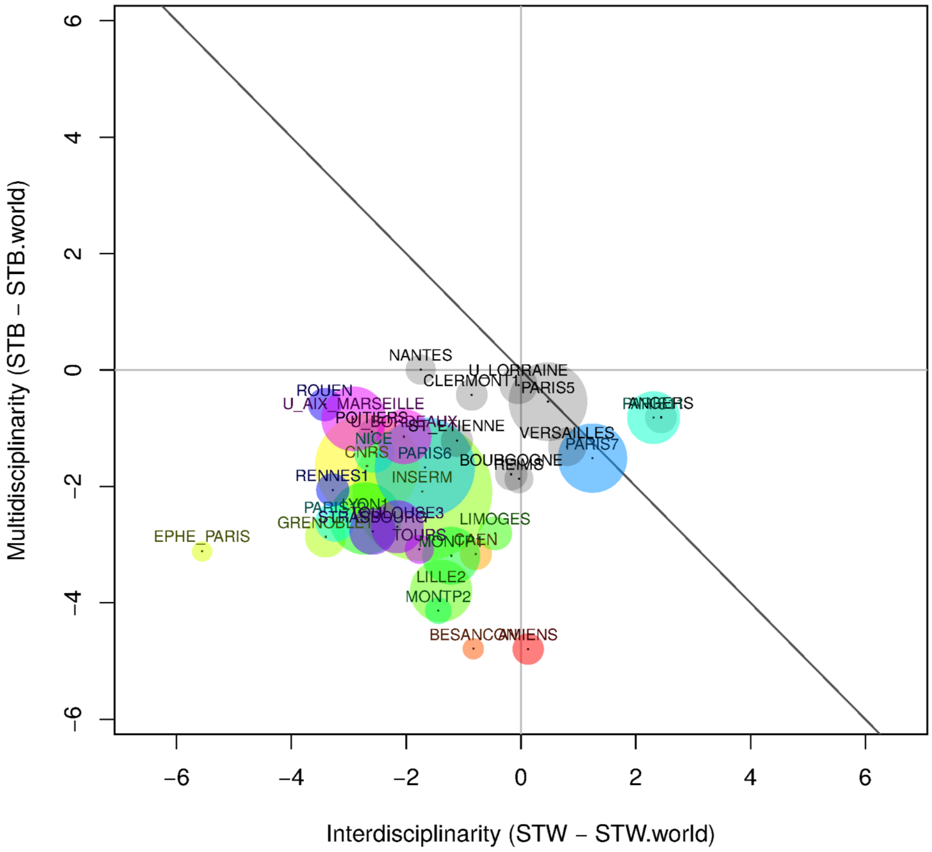

S2 Fig

NEUROSCIENCES (RU)

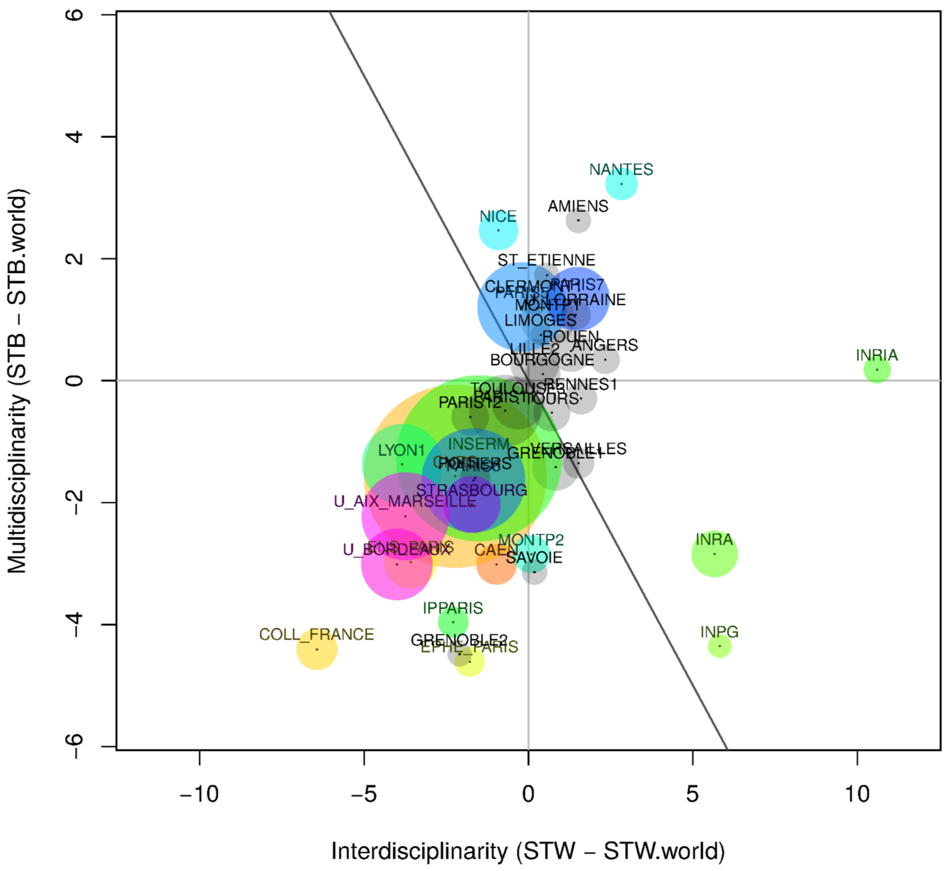

PHARMACOLOGY & PHARMACY (TU)

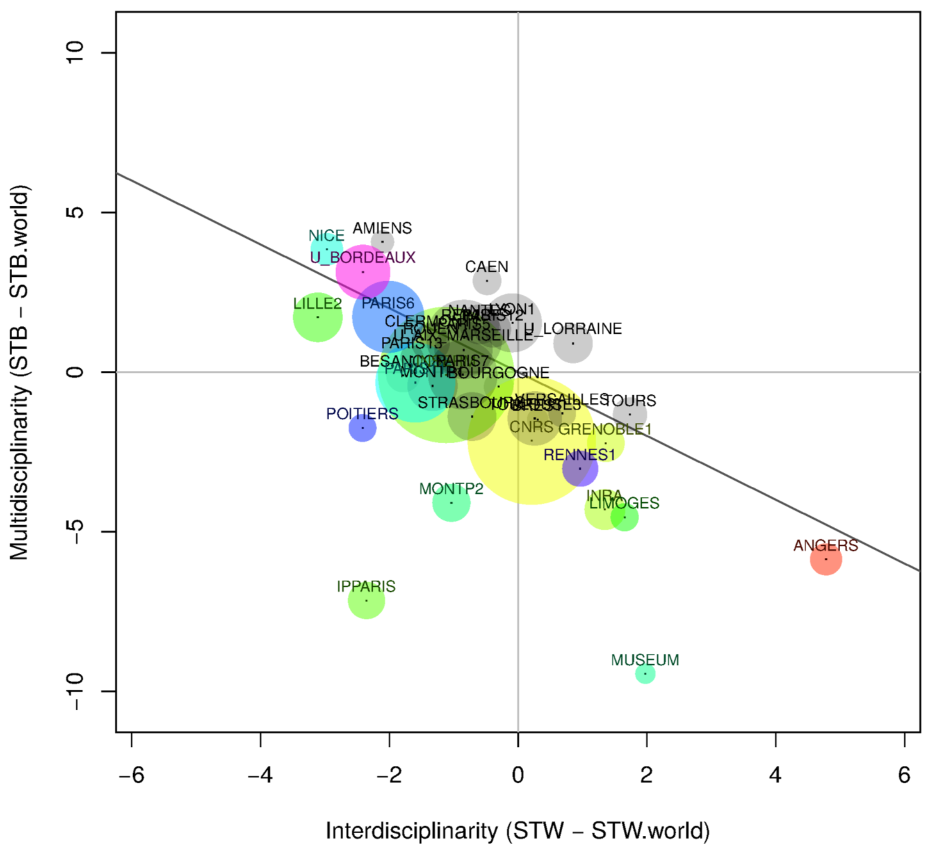

S2 Fig
