# Supplementary figures and images for: Analysing Institutions Interdisciplinarity by Extensive Use of Rao-Stirling Diversity Index
Source: PLoS One. 2017 Jan 23;12(1):e0170296. doi: 10.1371/journal.pone.0170296 (PMC5256946; doi:10.1371/journal.pone.0170296)

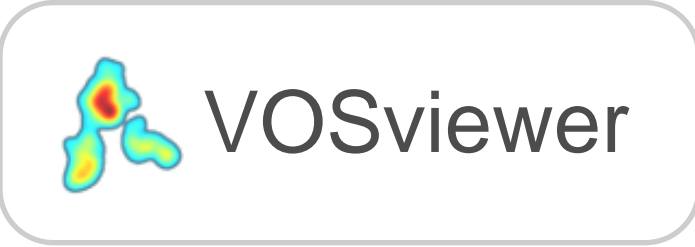

Supplement: S2 Fig — The networkfile is category similarity matrix.txt on the GitHub repository. Cluster resolution is 1.5 which provides 6 clusters. (PDF) [file pone.0170296.s004.pdf]

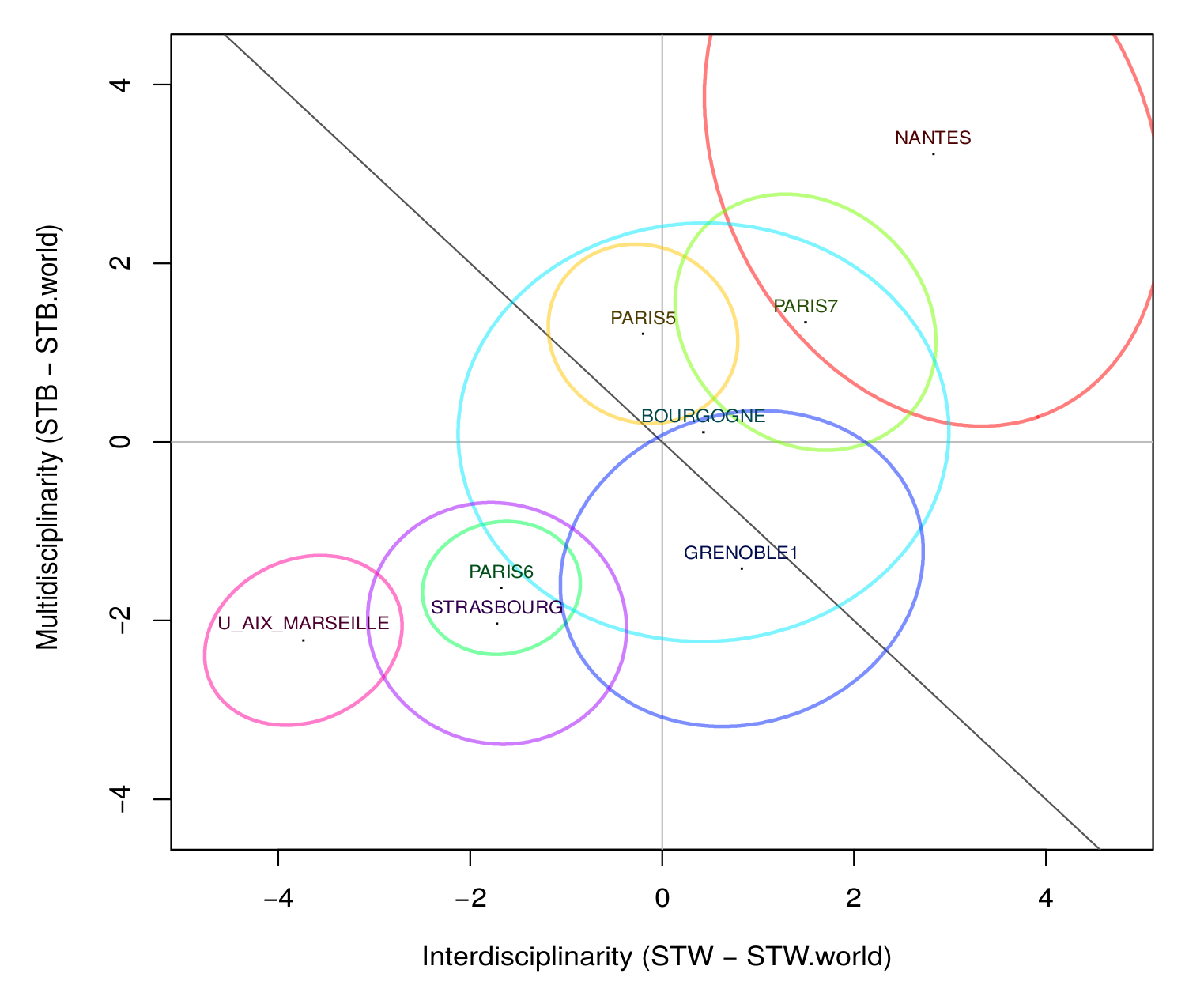

Supplement: S3 Fig — If an ellipsis does not cross the second bisector (i.e. the line x + y = 0), the overall indicator ST = STW + STB is significantly different from zero at level 0.02. If the ellipses of two universities do not overlap, their pairs (STW, STB) are significantly different at level 0.04. (TIFF) [file pone.0170296.s006.tiff]
